# Carotid Intima-Media Thickness and Long-Term Exposure to Traffic-Related Air Pollution in Middle-Aged Residents of Taiwan: A Cross-Sectional Study

**DOI:** 10.1289/ehp.1408553

**Published:** 2015-03-20

**Authors:** Ta-Chen Su, Juey-Jen Hwang, Yu-Cheng Shen, Chang-Chuan Chan

**Affiliations:** 1 Department of Internal Medicine and Cardiovascular Center, National Taiwan University Hospital, College of Medicine, National Taiwan University, Taipei, Taiwan; 2 Institute of Industrial Hygiene and Occupational Medicine, College of Public Health, National Taiwan University, Taipei, Taiwan

## Abstract

**Background:**

Associations between long-term exposure to air pollution and carotid intima-media thickness (CIMT) have inconsistent findings.

**Objectives:**

In this study we aimed to evaluate association between 1-year average exposure to traffic-related air pollution and CIMT in middle-aged adults in Asia.

**Methods:**

CIMT was measured in Taipei, Taiwan, between 2009 and 2011 in 689 volunteers 35–65 years of age who were recruited as the control subjects of an acute coronary heart disease cohort study. We applied land-use regression models developed by the European Study of Cohorts for Air Pollution Effects (ESCAPE) to estimate each subject’s 1-year average exposure to traffic-related air pollutants with particulate matter diameters ≤ 10 μm (PM_10_) and ≤ 2.5 μm (PM_2.5_) and the absorbance levels of PM_2.5_ (PM_2.5_abs), nitrogen dioxide (NO_2_), and nitrogen oxides (NO_x_) in the urban environment.

**Results:**

One-year average air pollution exposures were 44.21 ± 4.19 μg/m^3^ for PM_10_, 27.34 ± 5.12 μg/m^3^ for PM_2.5_, and (1.97 ± 0.36) × 10^–5^/m for PM_2.5_abs. Multivariate regression analyses showed average percentage increases in maximum left CIMT of 4.23% (95% CI: 0.32, 8.13) per 1.0 × 10^–5^/m increase in PM_2.5_abs; 3.72% (95% CI: 0.32, 7.11) per 10-μg/m^3^ increase in PM_10_; 2.81% (95% CI: 0.32, 5.31) per 20-μg/m^3^ increase in NO_2_; and 0.74% (95% CI: 0.08, 1.41) per 10-μg/m^3^ increase in NO_x_. The associations were not evident for right CIMT, and PM_2.5_ mass concentration was not associated with the outcomes.

**Conclusions:**

Long-term exposures to traffic-related air pollution of PM_2.5_abs, PM_10_, NO_2_, and NO_x_ were positively associated with subclinical atherosclerosis in middle-aged adults.

**Citation:**

Su TC, Hwang JJ, Shen YC, Chan CC. 2015. Carotid intima–media thickness and long-term exposure to traffic-related air pollution in middle-aged residents of Taiwan: a cross-sectional study. Environ Health Perspect 123:773–778; http://dx.doi.org/10.1289/ehp.1408553

## Introduction

An increasing number of cohort studies have shown that long-term exposure to air pollution is associated with all-cause and cardiovascular mortality (Hart et al. 2011; Laden et al. 2006; Yap et al. 2012; Yorifuji et al. 2013). A recent air pollution review completed by the American Heart Association suggested that the inhalation of particulate matter (PM) accelerates or enhances the development of atherosclerosis, and triggers clinical ischemic events (Brook et al. 2010).

Increased carotid intima-media thickness (CIMT) is an important indicator of subclinical atherosclerosis and provides a means to assess the development and progression of atherosclerotic vascular disease (O’Leary et al. 1999). The high burden of cardiovascular disease (CVD) and the extraordinarily high mortality rates associated with it indicate that CVD is a major public health concern in several countries. Furthermore, the process of global population aging indicates that this burden is likely to increase, necessitating more proactive CVD prevention strategies. Atherosclerosis begins early in life and progresses throughout the life span. The subclinical atherosclerosis index of CIMT increases with age and in line with age-dependent CVD risk factors (Su et al. 2012). CIMT has been used as an index of subclinical atherosclerosis and CVD in many epidemiological and clinical studies (Chien et al. 2008; Lorenz et al. 2007; O’Leary et al. 1999; Su et al. 2001, 2012).

Several studies examining the association between long-term exposure to air pollution and CIMT have reported inconsistent findings (Bauer et al. 2010; Breton et al. 2012; Künzli et al. 2005, 2010; Lenters et al. 2010; Tonne et al. 2012). One possible explanation for this inconsistency is the misclassification of individual exposures to air pollution due to the use of fixed-site monitoring data; the usual approach applied was to create a spatial model to describe the space between fixed-site monitors (Bauer et al. 2010; Breton et al. 2012; Künzli et al. 2005, 2010; Lenters et al. 2010; Tonne et al. 2012), or the distance to major roads to assign individual exposure (Bauer et al. 2010; Künzli et al. 2010; Lenters et al. 2010). A large-scale project conducted by the European Union called the European Study of Cohorts for Air Pollution Effects (ESCAPE) focused on the health effects of long-term exposure to air pollution in existing cohorts, with the aim of improving the estimation of pollution exposure levels of individuals within those cohorts (Beelen et al. 2014; Cesaroni et al. 2014; Eeftens et al. 2012a, 2012b; Raaschou-Nielsen et al. 2013).

This project integrated 24 research groups from 15 European countries. The National Taiwan University was the 25th partner to participate in ESCAPE (Eeftens et al. 2012a) and is the only ESCAPE partner based outside of Europe. ESCAPE used dedicated exposure assessment campaigns to monitor PM with diameter ≤ 10 μm or ≤ 2.5 μm (PM_10_ and PM_2.5_), and PM_2.5_ absorbance (PM_2.5_abs), nitrogen oxide (NO_2_), and NO_x_ (nitrogen oxides; NO and NO_2_). The study also obtained geographical information about land use in participating cities to develop land-use regression (LUR) models (Eeftens et al. 2012a, 2012b) and assigned 1-year exposures to air pollution to the cohort’s members. The ESCAPE project has reported significant associations of long-term air pollution effects with acute coronary events, lung cancer, and mortality by harmonizing air pollution exposures on study subjects across European cohorts (Beelen et al. 2014; Cesaroni et al. 2014; Raaschou-Nielsen et al. 2013).

The present study was carried out to evaluate the association between 1-year average exposures to air pollution and CIMT in adults living in the Taipei metropolis of Taiwan.

## Methods

*Study subjects.* Between 2009 and 2011, 689 adults 35–65 years of age attended a case–control study of work- and environment-related cardiovascular diseases as the control subjects of acute coronary heart diseases (CHD) in National Taiwan University Hospital (Cheng et al. 2014). The subjects were volunteers recruited by a bulletin announcement for participating in a case–control study of work- and environment-related cardiovascular diseases in the hospital. Participants reported they were free from physician-diagnosed CHD, cerebrovascular disease, and heart failure. Participants underwent noninvasive cardiovascular examination, including carotid duplex, blood sampling, and a structured questionnaire relating to lifestyle (smoking, alcohol use, and exercise habit), as well as medical history. After a fasting period of 10–14 hr, subjects who were not reported to be diabetic before enrollment underwent a standard oral glucose tolerance test with 75-g glucose loading. A fasting venous blood sample was sent for measurement of fasting blood glucose; lipid profile, including total cholesterol, triglycerides, high- and low-density lipoprotein cholesterol (HDL-C and LDL-C); albumin; renal function; and liver function. The institutional review board of the National Taiwan University Hospital ethics committee approved this study, and all participants gave informed, written consent before recruitment.

*Assessment of CIMT and carotid atherosclerosis.* Carotid atherosclerosis in the common carotid artery (CCA) was assessed by measuring CIMT using a high-resolution B-mode, GE Vivid *i* ultrasound system (GE Healthcare, Horten, Norway) equipped with a 3.5- to 10-MHz real-time B-mode scanner. The measurement protocol for carotid atherosclerosis has been described previously (Chien et al. 2008; Lin et al. 2013; Su et al. 2001, 2012). The CIMT measurement was a system-based built-in software in GE Vivid *i* ultrasound system. Maximum and mean CIMT distal to the carotid bifurcation, bulb, and internal carotid artery were measured bilaterally. CCA1 and CCA2 are points located at 0–1 cm and 1–2 cm, respectively, along the CCA, distal to the carotid bifurcation. CIMT of the posterior wall of the distal CCA was measured as the distance from the leading edge of the first echogenic line to the second line. All scans were recorded on a digitalized memory system in Digital Imaging and Communications in Medicine format for subsequent offline analysis. The digitized M-mode was later analyzed offline using a computer program, in which each image was magnified, and the CIMT between two successive R waves was measured by automated analysis software (GE Healthcare).

Because measurements of CIMT taken in the CCA are more strongly associated with cardiovascular risk factors than CIMT in the bulb and internal carotid artery (Polak et al. 2010), we chose CIMT in the CCA as our outcome measure for this study. The technician conducted a second reading for 30 randomly selected participants 2 weeks after the first measurement was taken. The reliability of mean and maximum CIMT measurement in the left and right CCA had an excellent intraobserver agreement of 98.8% and 97.6% for LCCA, and 97.8% and 97.3% for RCCA, respectively.

*Assessments of vascular risk factors.* We recruited volunteers to receive cardiovascular health examination in the morning of Wednesday or Saturday during 0830–1200 hours. Subjects who had systolic blood pressure (SBP) ≥ 140 mmHg and/or diastolic blood pressure ≥ 90 mmHg, or were taking antihypertensive medicines, were considered to be hypertensive. Diabetes mellitus (DM) was defined as fasting glucose levels ≥ 126 mg/dL (7.0 mmol/L), a history of DM controlled by medication, or DM diagnosed by the oral glucose tolerance test [glucose level at 120 min postchallenge ≥ 200 mg/dL (11.1 mmol/L)]. Hypercholesterolemia was defined as a cholesterol level ≥ 240 mg/dL (6.2 mmol/L) or being treated with lipid-lowering medications. Obesity was defined as body mass index (BMI) ≥ 27 kg/m^2^, according to the Taiwanese definition (Pan et al. 2004).

*Exposure to PM_10_, PM_2.5_, PM_2.5_abs, NO_2_, and NO_x_.* Exposure measurements for each subject in Taipei were made according to a verified methodology developed by the ESCAPE project that has been described elsewhere (Lee et al. 2014). Briefly, air pollution monitoring campaigns were performed between September 2009 and August 2010 in the study area. Three 2-week measurements of NO_2_ and NO_x_ were performed within 1 year at 40 sites. Simultaneous measurements of PM_10_, PM_2.5_, and PM_2.5_abs (blackness of the PM_2.5_ exposed filter, determined by measurement of light reflectance as a marker for soot and black carbon) were performed at half of the sites. Results from the three measurements were averaged to estimate the annual average concentration of each pollutant. Variables on nearby traffic, population/household density, and land use derived from geographic information systems (GIS) were evaluated as predictors of the spatial variation in annual average concentrations. The traffic representations of Taipei were obtained from a digital map of traffic networks in Taiwan, which was completed by the Institute of Traffic and Transportation in 2010. Population and household data for 2010 were collected from the Household Registration Office of each district in Taipei. The topographic altitude map was obtained from the City Offices. Land use information was taken from the Land Use Investigation of Taiwan conducted by the National Land Surveying and Mapping Center in 2007 (http://www.nlsc.gov.tw/En/MakePage/166?level=166). We required a spatial resolution of at least 100 m. Regression models were developed to maximize the adjusted explained variance, using a supervised forward stepwise approach. LUR models were then used to estimate annual average air pollution concentrations at the participants’ addresses, for which the same GIS predictor variables were collected. Overall model performance was evaluated by leave-one-out cross-validation: Each site was sequentially left out from the model while the included variables were left unchanged.

*Statistical analysis.* For the basic characteristics of participants, continuous variables are expressed as mean ± SD, and binary variables are presented as number (percent). The 1-year average long-term exposure concentrations for the five air pollutants (PM_2.5_, PM_2.5_abs, PM_10_, NO_2_, and NO_x_) are expressed as mean (± SD), median, and interquartile range. The rationale for choosing covariates in the multivariate analysis was based on prior knowledge of major risk factors for CVD and atherosclerosis, such as hypertensive status (or SBP), hypercholesterolemia (or LDL-C), DM, or obesity. In addition, age, sex, education, ever having smoked, and lipid-lowering treatment are also important factors that may affect atherosclerosis.

Multivariate linear regression analyses were used to assess the association between maximum and mean CIMT at LCCA and RCCA and for both combined (CCA) in association with air pollution exposures. All models were adjusted for age, BMI, SBP, and serum LDL-C concentration as continuous variables; and sex, DM (yes/no), education (≥ or < 12 years), and ever (vs. never) having smoked as dichotomous variables. Associations with the five pollutants studied were expressed as percentage change [95% confidence interval (CI)] in estimated maximum and mean CIMT at CCA per selected unit increase in 1-year average level of each air pollutant, as estimated by LUR models. For comparison, the unit of air pollutants was selected in line with the ESCAPE project.

Interaction analysis was conducted for evaluating whether the covariates are confounders. Subgroup analysis was conducted for evaluating effect modification for air pollution by stratified age, sex, BMI, hypertension, hypercholesterolemia, lipid-lowering treatment, DM, and ever smoking. When the single-pollutant model is significant, we will do two-pollutant model. In this study, we applied PM_2.5_abs as index of particle pollutant and NO_2_ as index of gaseous pollutant to test the interaction effects based on the major findings.

All analyses were performed using SAS software (version 9.1.3; SAS Institute Inc., Cary, NC). A significance level of 0.05 with a two-tailed distribution was considered to be statistically significant.

## Results

The basic characteristics and exposure data for the 689 study subjects are listed in Table 1. Our study population was composed mainly of males (72%) and middle-aged adults with a mean age of 49 ± 8 and 52 ± 8 years for males and females, respectively. Mean and maximum CIMT of the LCCA were 0.59 ± 0.13 mm and 0.73 ± 0.15 mm for males and 0.57 ± 0.12 mm and 0.72 ± 0.17 mm for females, respectively. The mean and maximum CIMT of the RCCA were 0.58 ± 0.12 mm and 0.72 ± 0.15 mm for males and 0.56 ± 0.10 mm and 0.69 ± 0.11 mm for females, respectively. The study population had low levels of ever smoking (32.9% of males and 1.6% of females had ever smoked), and a minority had < 12 year formal education (21% of males and 31% of females). The prevalence of DM was 34% for males and 35% for females. The prevalence of hypercholesterolemia was 41% for males and 52% for females in the study population.

**Table 1:** Demographic and clinical characteristics and CIMT in the CCA of participants.

Variable	Male (*n* = 497)	Female (*n* = 192)	*p*-Value
Age (years)	49 ± 8	52 ± 8	< 0.001
BMI (kg/m^2^)	25.6 ± 3.0	23.6 ± 4.0	< 0.001
Systolic blood pressure (mmHg)	125.0 ± 13.9	124.0 ± 15.7	0.42
Diastolic blood pressure (mmHg)	77.6 ± 9.6	76.4 ± 10.3	0.16
Hypertension, yes	147 (29.58)	58 (30.21)	0.86
Cholesterol (mmol/L)	6.0 ± 1.5	6.6 ± 2.0	< 0.001
Triglycerides (mmol/L)	2.7 ± 3.1	2.7 ± 3.4	0.79
Hypercholesterolemia^*a*^, yes	203 (40.8)	99 (51.6)	0.01
HDL-C (mmol/L)	1.3 ± 0.4	1.6 ± 0.5	< 0.001
LDL-C (mmol/L)	3.6 ± 1.3	3.7 ± 1.7	0.63
Fasting blood glucose (mmol/L)	5.5 ± 1.4	5.3 ± 1.1	0.10
DM^*b*^, yes	169 (34.0)	68 (35.2)	0.71
Ever smoking habit, yes	163 (32.8)	3 (1.6)	< 0.001
Current smoker	108 (21.69)	2 (1.04)	< 0.001
Education, < 12 years	106 (21.33)	60 (31.3)	0.006
Lipid-lowering treatment	51 (10.26)	13 (6.77)	0.16
CIMT (mm)
CCA, mean	0.58 ± 0.11	0.56 ± 0.10	0.04
Maximum	0.73 ± 0.14	0.70 ± 0.12	0.06
LCCA, mean	0.59 ± 0.13	0.57 ± 0.12	0.09
Maximum	0.73 ± 0.15	0.72 ± 0.17	0.30
RCCA, mean	0.58 ± 0.12	0.56 ± 0.10	0.04
Maximum	0.72 ± 0.15	0.69 ± 0.11	0.02
Values are mean ± SD or *n* (%). ^***a***^Cholesterol level ≥ 6.2 mmol/L or being treated with lipid-lowering medications. ^***b***^Fasting glucose levels ≥ 7.0 mmol/L, a history of DM controlled by medication, or DM diagnosed by the oral glucose tolerance test (glucose level at 120 min postchallenge ≥ 11.1 mmol/L).			

There were four to six variables, such as the length of major road, road area, urban green area, industrial area, commercial area, and river area, included in the LUR models. The leave-one-out cross-validation *R*^2^ of LUR models used for exposure estimation were 0.91, 0.92, 0.74, 0.63, and 0.81 for PM_2.5_, PM_2.5_abs, PM_10_, NO_2_, and NO_x_, respectively. The root mean square errors of the above air pollutants were 1.51 μg/m^3^, 0.09 × 10^–5^/m, 2.36 μg/m^3^, 6.36 μg/m^3^, and 10.7 μg/m^3^, respectively. LUR estimated 1-year average air pollution levels indicate that our study subjects were exposed to relatively high concentrations of PM_2.5_ (27.34 ± 5.12 μg/m^3^) and PM_10_ (44.21 ± 4.19 μg/m^3^) compared to the air quality goals of the World Health Organization (10 μg/m^3^ for PM_2.5_ and 20 μg/m^3^ for PM_10_) (http://www.who.int/mediacentre/factsheets/fs313/en/). In addition, the estimated exposures of PM_2.5_abs, NO_2_, and NO_x_ were 1.97 ± 0.36 (10^–5^/m), 46.13 ± 11.32 μg/m^3^, and 68.37 ± 21.35, respectively (Table 2). A correlation analysis found positive correlations between each of the exposures among these five air pollutants for our study subjects, with correlation coefficients ranging from 0.38 to 0.91 (see Supplemental Material, Table S1).

**Table 2:** Estimated 1-year air pollution concentration by land-use regression models.

Variables	Mean ± SD	Median	Q1	Q3
PM_2.5_ (μg/m^3^)	27.34 ± 5.12	26.98	23.67	30.45
PM_2.5_abs (10^–5^/m)	1.97 ± 0.36	1.96	1.70	2.23
PM_10_ (μg/m^3^)	44.21 ± 4.19	43.56	40.93	46.92
NO_2_ (μg/m^3^)	46.13 ± 11.32	46.53	40.40	53.62
NO_x_ (μg/m^3^)	68.37 ± 21.35	67.68	57.01	83.45
Q, quartile.				

After adjusting for the *a priori* covariates, maximum CIMT at LCCA was significantly higher in association with average 1-year exposures to PM_10_, PM_2.5_abs, NO_2_, and NO_x_, but not with PM_2.5_ (Table 3). The average percentage difference in maximum CIMT at LCCA was 4.23% higher (95% CI: 0.32, 8.13) per 1 × 10^–5^/m PM_2.5_abs, 3.72% higher (95% CI: 0.32, 7.11) per 10 μg/m^3^ PM_10_, 2.81% higher (95% CI: 0.32, 5.31) per 20 μg/m^3^ NO_2_, and 0.74% higher (95% CI: 0.08, 1.41) per 10 μg/m^3^ NO_x_. Mean CIMT at LCCA was significantly higher in association with exposure to PM_10_ and PM_2.5_abs only (Table 3). PM_2.5_ mass was not associated with maximum or mean CIMT at LCCA, and none of the air pollutants were associated with maximum or mean CIMT at right CCA. However, in the association between air pollutants and combined CCA estimates, only NO_2_ was evident with maximum CIMT and PM_10_ was significantly with mean CIMT. Both left and right maximum and mean CIMT were significantly associated with established cardiovascular risk factors. For example, maximum and mean LCCA in models that also included PM_2.5_abs were higher in association with LDL-C (1.80% higher, 95% CI: 0.79, 2.81 for a 1-mmol/L increase), SBP (2.61% higher, 95% CI: 1.56, 3.66 for a 10-mmHg increase), age (1.11% higher, 95% CI: 0.92, 1.31 for a 1-year increase), and DM (4.06% higher, 95% CI: 0.84, 7.28 for DM vs. no DM). Ever versus never smoking was positively but not significantly associated with maximum and mean CIMT at LCCA (1.60% higher; 95% CI: –1.90, 5.10 and 1.38% higher; 95% CI: –2.04, 4.80), respectively. Associations between model covariates and RCCA were consistent with those for LCCA (data not shown). One example of such findings for PM_2.5_abs and NO_2_ is presented in Supplemental Material, Table S2. The associations of PM_2.5_abs, PM_10_, NO_2_, and NO_x_ with CIMT became insignificant in two-pollutant models.

**Table 3:** Multivariate linear regression analyses for percent difference (95% CI) in maximum and mean CIMT at LCCA and RCCA and for both combined (CCA) in association with air pollution exposures.

Exposure (exposure increment)	CIMT at LCCA	CIMT at RCCA	Combined CIMT
Maximum CIMT
PM_2.5_abs (10^–5^/m)	4.23 (0.32, 8.13)*	1.63 (–1.98, 5.23)	2.94 (–0.25, 6.14)
PM_2.5_ (5 μg/m^3^)	–0.28 (–1.68, 1.12)	–0.30 (–1.00, 1.58)	0.001 (–1.15, 1.15)
PM_10_ (10 μg/m^3^)	3.72 (0.32, 7.11)*	1.59 (–1.54, 4.72)	2.66 (–0.11, 5.44)
NO_2_ (20 μg/m^3^)	2.81 (0.32, 5.31)*	1.79 (–0.52, 4.09)	2.31 (0.26, 4.35)*
NO_x_ (10 μg/m^3^)	0.74 (0.08, 1.41)*	0.29 (–0.32, 0.90)	0.52 (–0.02, 1.06)
Mean CIMT
PM_2.5_abs (10^–5^/m)	4.19 (0.38, 8.00)*	0.61 (–2.41, 5.11)	2.78 (–0.50, 6.07)
PM_2.5_ (5 μg/m^3^)	–0.27 (–1.64, 1.10)	0.16 (–1.19, 1.51)	–0.05 (–1.23, 1.12)
PM_10_ (10 μg/m^3^)	3.58 (0.27, 6.89)*	1.76 (–1.51, 5.02)	2.68 (0.17, 5.53)*
NO_2_ (20 μg/m^3^)	1.98 (–0.46, 4.42)	1.63 (–0.77, 4.03)	1.81 (–0.30, 3.91)
NO_x_ (10 μg/m^3^)	0.51 (–0.14, 1.16)	0.16 (–0.48, 0.80)	0.34 (–0.22, 0.90)
Adjusted for age, sex, BMI, SBP, LDL-C, lipid-lowering treatment (yes/no), DM (yes/no), education (< or ≥ 12 years), and ever versus never smoking history. *p < 0.05.			

We applied a stratification approach to test differences in subgroups and added interaction terms in our models to test for interactions between air pollutants and key cardiovascular risk factors. The results of effect modification by subgroup analysis showed positive associations between 1-year PM_2.5_abs air pollution exposure and CIMT at LCCA among female and subjects without hypercholesterolemia, never smokers, and non-obese (see Supplemental Material, Table S3). We also found positive associations between 1-year NO_2_ exposure and CIMT at LCCA among female participants and subjects without hypertension or hypercholesterolemia. In addition, subjects with age ≥ 50 years, non-obese or without DM also were sensitive groups (see Supplemental Material, Table S4).

We performed the sensitivity analyses with models by including the eight interaction terms (age, sex, hypertension, BMI, hypercholesterolemia, DM, smoking, and lipid-lowering treatment) one by one, respectively. However, the main air pollution effects remained stable in the analyses for seven of the eight covariates except age in our models. Aged subjects have a positive correlation coefficient of interaction with NO_2_ levels (NO_2_ × age) for CIMT of LCCA (see Supplemental Material, Tables S3 and S4).

## Discussion

This study demonstrates the positive association between long-term air pollution exposure, particularly PM (PM_2.5_abs and PM_10_), NO_2_, and NO_x_, and subclinical atherosclerosis in terms of CIMT among adults 35–65 years of age in the Taipei metropolis. The strength of the study was that it yielded consistent findings for the associations between four of five monitored air pollutants and maximum CIMT at LCCA after controlling for other major cardiovascular risk factors.

There are several limitations in this study. We cannot rule out that the results might be overestimated or underestimated. The reliance on volunteers is a major limitation in this study, which may limit the generalization of the study findings. Using the present contrasts in pollution instead of the true ones from the past, associations can potentially be inflated. According to the annual report of Taiwan Environmental Protection Administration (TEPA), air pollution levels in Taipei showed relatively stable PM_10_ concentrations but gradually decreasing NO_2_ levels during 1997–2009 (TEPA 2013). The lack of data on time–activity for each individual may have led to some misclassification of the amount of exposure during working hours and during other activities undertaken outside of the home (Kaufman et al. 2012). Current LUR models may not represent true exposure for subjects living in high-rise housing in our study, which should be addressed by additional spatial modeling in future studies. Another limitation of the study is the lack of consideration of other risk factors, such as genetic and other environmental factors that might predispose individuals to atherosclerosis (Bhatnagar 2006; Kaufman 2010; Lin et al. 2013). The use of education data only as an indicator for socioeconomic status (SES) may not fully control for the effect of SES on CIMT. There can be a high correlation among places, income, and CIMT in Taipei, which needs to be explored by better measures/proxy of SES in future studies.

This investigation reconfirms the findings of previous studies in the United States and Europe; long-term exposure to PM_10_ and/or PM_2.5_ air pollution is associated with CIMT (Bauer et al. 2010; Chen et al. 2010; Künzli et al. 2005; Lenters et al. 2010). Recent prospective studies also have demonstrated this association (Bauer et al. 2010; Breton et al. 2012; Künzli et al. 2010; Tonne et al. 2012). Long-term exposures to NO_2_ and NO_x_ were associated with significantly higher maximum CIMT at LCCA, which supports a role of traffic-related gaseous air pollution. Furthermore, PM_2.5_abs, but not PM_2.5_ mass concentration, was associated with subclinical atherosclerosis in our ethnic Chinese population. And, this is the first study to use the LUR models developed by ESCAPE to estimate residential long-term average air pollution levels and successfully link them to individual CIMT in Asia. All of the LUR models for PM_2.5_, PM_2.5_abs, PM_10_, NO_2_, and NO_x_ had high leave-one-out cross-validation *R*^2^ (0.63–0.92), suggesting that the LUR models used for exposure estimation explained the variance of these pollutants in the study area.

The size of the smoking effect on CIMT, though not significant, is comparable with our previous community-based study in Taipei, which did not include air pollution effects (Su et al. 2012). However, we cannot infer that smoking contributes less to subclinical atherosclerosis than does air pollution. First, ever smoking including those currently smoking or ever have smoked who quit for many years may underestimate the atherogenic effects coming from current smoking. Second, we did not quantify the pack-years, which may underestimate the significant effects coming from heavy smokers with ≥ 10 pack-years. Third, the estimated 1-year air pollution concentrations in Taipei area were significantly higher than those measured in most areas in the United States and the European Union; thus the relatively higher contribution of air pollution on CIMT might attenuate the partly similar pathogenic effects from ever smoking observed in this study.

The subgroups that are otherwise at low risk of CVD might be more susceptible to PM_2.5_abs and NO_2_ air pollution exposure; however, the association with NO_2_ exposure was stronger among those ≥ 50 years of age compared with younger participants. As for the modification effect of PM_2.5_abs, the size of point estimate for those taking lipid-lowering treatment was larger than for those who were not. However, the sensitivity analyses revealed no significant interaction effect on the risk for CIMT among these seven major CVD risk factors and air pollutants respectively. Our findings are partly in agreement with the results of Künzli et al. (2005), who found that those on statins treatment, aged, women, and nonsmokers were more susceptible to air pollution. However, our study showed the association among those taking lipid-lowering treatment was stronger for PM_2.5_abs but weaker for NO_2_; the interactions were not significant in either case, and our study population had small numbers on treatment. Our subgroup analysis also was not consistent with findings in the Heinz Nixdorf Recall (HNR) study, which identified the association was slightly stronger in younger and obese subjects (Bauer et al. 2010). Associations with hypercholesterolemia may be attenuated by adjustment for treatment with lipid-lowering medications. Because of the limited precision of the estimates, we must interpret these subgroup effects with caution.

The atherosclerotic process accelerates generally on both sides in the presence of traditional risk factors such as hypercholesterolemia, DM, higher BP, male sex, and older age. Our results also underscore the importance of measuring both the left and right CCAs for assessing the association with air pollution exposure. We found a stronger association between the four pollutants and maximum LCCA compared with maximum RCCA, but only two pollutants had a stronger association with mean LCCA compared with mean RCCA. It would be reasonable to suggest briefly that there may be a physiologic basis, but there is very little prior evidence to support this. We need further study to confirm it.

The fact that the black carbon content of PM_2.5_abs has a similar association with CIMT compared to that of PM_10_ mass, as demonstrated here, supports the potential importance of composition or components of PM_2.5_ in cardiovascular health (Wu et al. 2012). The main sources of black carbon are combustion engines, particularly diesel engines in the Taipei metropolis. An assessment of the health risks and the establishment of regulations for PM_2.5_abs are warranted.

The mechanisms linking air pollution and CVD are proposed in the scientific statement from the American Heart Association by Brook et al. (2010), and our previous studies may add support for these mechanisms (Chen et al. 2014; Chuang et al. 2007; Wu et al. 2010, 2012). Seasonal variation in PM_10_ levels predicted seasonal variations in plasma fibrinogen levels in a panel study of middle-aged adults (Su et al. 2011b). In addition, this study provides supporting evidence of an increased risk of atherosclerosis following long-term air pollution exposure in Asia.

Further investigation is necessary to assess the atherogenic effects of the high concentrations of air pollutants in Taipei, such as the estimated average levels of PM_2.5_, PM_10_, and NO_2_, which are twice to three times those observed in the United States and Europe. Because the urban areas that suffer most from air pollution in the world are located in Asian countries such as China and India (Su et al. 2011a), the emerging data regarding adverse cardiovascular effects in Taiwan should be urgently considered by Asian countries. For the proactive prevention of air pollution-related CVD, the catastrophic episode of smog in Beijing, China, in January–February 2013 shows that action must be taken as soon as possible (Ouyang 2013).

In conclusion, long-term exposure to traffic-related air pollution with PM_2.5_abs, PM_10_, NO_2_, and NO_x_ was positively associated with subclinical atherosclerosis, represented by maximal CIMT in middle-aged adults. But the associations were not evident for right CIMT, and PM_2.5_ mass concentration was not associated with the outcomes.

## Supplemental Material

(268 KB) PDF
