# Supplementary material for: Carotid Intima-Media Thickness and Long-Term Exposure to Traffic-Related Air Pollution in Middle-Aged Residents of Taiwan: A Cross-Sectional Study
Source: Environ Health Perspect. 2015 Mar 20;123(8):773–8. doi: 10.1289/ehp.1408553 (PMC4529007; doi:10.1289/ehp.1408553)
Supplement: (268 KB) PDF [file ehp.1408553.s001.acco.pdf]

Environ Health Perspect
doi: 10.1289/ehp.1408553

Note to Readers: ***EHP*** **strives to ensure that all journal content is accessible to all readers. However, some figures and Supplemental Material published in *EHP* articles may not conform to 508 standards due to the complexity of the information being presented. If you need assistance accessing journal content, please contact ehp508@niehs.nih.gov. Our staff will work with you to assess and meet your accessibility needs within 3 working days.**

Supplemental Material

Carotid Intima–Media Thickness and Long-Term Exposure to Traffic-Related Air Pollution in Middle-Aged Residents of Taiwan: A Cross-Sectional Study

Ta-Chen Su, Juey-Jen Hwang, Yu-Cheng Shen, and Chang-Chuan Chan

Table of Contents

Table S1. Pearson's correlation between 5 different pollutants (N=689).

Table S2. Models for two pollutants ($PM_{2.5}$ abs and NO_2) as determinants of carotid intima-media thickness (CIMT) after adjusting other cardiovascular covariates.

Table S3. Interaction effects and the subgroups analysis to maximum CIMT at LCCA with $PM_{2.5}$ abs.

Table S4. Interaction effects and the subgroups analysis to maximum CIMT at LCCA with NO_2.

Table S1. Pearson's correlation between 5 different pollutants (N=689).

Exposure	Estimate	$PM_{2.5}$	$PM_{2.5abs}$	PM_{10}	NO_2	NO_x
$PM_{2.5}$	r	1.00	0.57	0.56	0.38	0.44
	P		<.001	<.001	<.001	<.001
$PM_{2.5}$ abs	r	0.57	1.00	0.56	0.55	0.56
	P	<.001		<.001	<.001	<.001
PM_{10}	r	0.56	0.56	1.00	0.46	0.44
	P	<.001	<.001		<.001	<.001
NO_2	r	0.38	0.55	0.46	1.00	0.91
	P	<.001	<.001	<.001		<.001
NO_x	r	0.44	0.56	0.44	0.91	1.00
	P	<.001	<.001	<.0001	<.001	

Table S2. Models for two pollutants ($PM_{2.5}$ abs and NO_2) as determinants of carotid intima-media thickness (CIMT) after adjusting other cardiovascular covariates.

Outcome	PM_{25}abs (10^{-5} m^{-1}) % difference (95% CI)	NO_2 (20 μg/m^3) % difference (95% CI)
Mean CIMT at LCCA	3.58 (-1.02, 8.18)	0.35 (-1.12, 1.82)
Mean CIMT at RCCA	-0.10 (-4.64, 4.43)	0.83 (-0.62, 2.28)
Combined mean CIMT	1.75 (-2.21, 5.72)	0.59 (-0.68, 1.86)
Maximum CIMT at LCCA	2.57 (-2.14, 7.28)	0.95 (-0.56, 2.45)
Maximum CIMT at RCCA	0.09 (-4.26, 4.44)	0.88 (-0.52, 2.27)
Combined maximum CIMT	1.35 (-2.50, 5.20)	0.91 (-0.32, 2.14)

Adjusted for age, male gender, body mass index, systolic blood pressure, LDL-cholesterol, lipid-lowering treatment, diabetes mellitus, education, and ever smoking history.

Table S3. Interaction effects and the subgroups analysis to maximum CIMT at LCCA with $PM_{2.5}$ abs.

Interaction Effect[a]		Subgroups Analysis[b]		
**Interaction Term**	**% difference (95% CI)**	**Subgroup**	**N**	**% difference (95% CI)**
$PM_{2.5}$ abs*Age	0.16 (-0.35, 0.68)	Age<50 years	371	1.18 (-1.01, 3.38)
		Age≥50 years	318	1.88 (-2.21, 5.97)
$PM_{2.5}$ abs*Male gender	-9.36 (-18.13, -0.60)	Female	192	4.98 (0.20, 9.76)*
		Male	497	1.05 (-1.24, 3.33)
$PM_{2.5}$ abs*Hypertension	0.06 (-0.21, 0.34)	Hypertension, No	484	1.80 (-0.48, 4.08)
		Hypertension, Yes	205	4.18 (-0.54, 8.89)
$PM_{2.5}$ abs*BMI	-0.44 (-1.69, 0.81)	BMI<27	531	2.54 (0.18, 4.89)*
		BMI≥27	158	2.08 (-2.35, 6.52)
$PM_{2.5}$ abs*Diabetes mellitus	2.03 (-6.36, 10.42)	Diabetes mellitus, No	452	1.86 (-0.20, 3.93)
		Diabetes mellitus, Yes	237	3.23 (-1.52, 7.98)
$PM_{2.5}$ abs*Ever smoking	-5.48 (-14.61, 3.64)	Ever Smoking, No	523	2.85 (0.38, 5.32)*
		Ever Smoking, Yes	166	0.08 (-3.73, 3.90)
$PM_{2.5}$ abs*Lipid-lowering treatment	-1.12 (-4.03, 1.79)	Lipid-lowering treatment, No	625	1.85 (-0.36, 4.07)
		Lipid-lowering treatment, Yes	64	3.53 (-2.60, 9.66)
$PM_{2.5}$ abs*Hypercholesterolemia	7.27 (-6.22, 20.75)	Hypercholesterolemia, No	388	3.28 (0.52, 6.03)*
		Hypercholesterolemia, Yes	301	1.32 (-1.85, 4.48)

[a]Multivariate linear regression analysis are with interaction term. [b]Multivariate linear regression analysis are conducted in subgroup. Both model are adjusted for age, male gender, body mass index, systolic blood pressure, LDL-cholesterol, lipid-lowering treatment, diabetes mellitus, education, and ever smoking history. Exposure increment used for calculation of percent change PM25abs in every 10^{-5} m^{-1}.

*$P < 0.05$.

Table S4. Interaction effects and the subgroups analysis to maximum CIMT at LCCA with NO_2.

Interaction Effect[a]		Subgroups Analysis[b]		
**Interaction Term**	**% difference (95% CI)**	**Subgroup**	**N**	**% difference (95% CI)**
NO_2*Age	0.40 (0.08, 0.72)*	Age<50 years	371	0.04 (-1.35, 1.43)
		Age≥50 years	318	3.13 (0.45, 5.80)*
NO_2*Male gender	-4.95 (-10.53, 0.63)	Female	192	2.90 (0.04, 5.77)*
		Male	497	0.69 (-0.79, 2.18)
NO_2*Hypertension	0.0 1(-0.18,0.19)	Hypertension, No	484	1.83 (0.38, 3.29)*
		Hypertension, Yes	205	0.92 (-2.08, 3.91)
NO_2*BMI	-0.35 (-1.17, 0.48)	BMI<27	531	1.62 (0.11, 3.12)*
		BMI≥27	158	1.16 (-1.73, 4.06)
NO_2*Diabetes mellitus	-0.51 (-5.86, 4.85)	Diabetes mellitus, No	452	1.73 (0.40, 3.07)*
		Diabetes mellitus, Yes	237	1.03 (-1.98, 4.04)
NO_2*Ever smoking	-1.81 (-7.46, 3.83)	Ever Smoking, No	523	1.75 (0.15, 3.34)*
		Ever Smoking, Yes	166	0.80 (-1.52, 3.11)
NO_2*Lipid-lowering treatment	-1.59 (-3.29, 0.11)	Lipid-lowering treatment, No	625	1.64 (0.23, 3.06)*
		Lipid-lowering treatment, Yes	64	-0.14 (-3.91, 3.62)
NO_2*Hypercholesterolemia	-2.70 (-11.16, 5.76)	Hypercholesterolemia, No	388	1.80 (0.08, 3.52)*
		Hypercholesterolemia, Yes	301	0.97 (-1.11, 3.06)

[a]Multivariate linear regression analysis are with interaction term. [b]Multivariate linear regression analysis are conducted in subgroup.

Adjusted for age, male gender, body mass index, systolic blood pressure, LDL-cholesterol, lipid-lowering treatment, diabetes mellitus, education, and ever smoking history. Exposure increment used for calculation of percent change NO_2 in every 20 $\mu g/m^3$.

*$P < 0.05$.
